# Identification of Group B Streptococcus Serotypes and Genotypes in Late Pregnant Women and Neonates That Are Associated With Neonatal Early-Onset Infection in a South China Population

**DOI:** 10.3389/fped.2020.00265

**Published:** 2020-05-27

**Authors:** Zhu Yao, Wu Jiayin, Zheng Xinyi, Chen Ling, He Mingyuan, Ma Simin, Lin Yayin, Lin Xinzhu, Chen Chao

**Affiliations:** ^1^Department of Neonatology, Women and Children's Hospital of Xiamen University, Xiamen, China; ^2^Department of Clinical Laboratory, Women and Children's Hospital of Xiamen University, Xiamen, China; ^3^School of Public Health, Xiamen University, Xiamen, China; ^4^Department of Neonatology, Children's Hospital of Fudan University, Shanghai, China

**Keywords:** group B *Streptococcus*, late pregnancy, early-onset infection, serotype, genotype

## Abstract

**Background:** Group B *streptococcus* (GBS) colonized in late pregnancies has been associated with neonatal early-onset GBS disease (GBS-EOD) in China.

**Objective:** This study investigated if GBS serotype and genotype in late pregnancy is associated with GBS-EOD, providing a reference for GBS-EOD prevention and treatment.

**Methods:** A total of 298 pregnant women with GBS colonization during their late pregnancy and 32 invasive GBS-EOD cases were included in this study for GBS serotyping and genotyping using commercial kits and DNA sequencing.

**Results:** We identified 266 GBS strains from mothers whose newborns were not infected with GBS-EOD. Serotype III [54.9% (146/266)] was the most common serotype, followed by Ib [17.3% (46/266)] and V [10.1% (27/266)]. ST19 was the most prevalent genotype [19.9% (53/266)], followed by ST862 [9.4% (25/266)] and ST12 [7.9% (21/266)]. We found that 32 mothers and their neonates with GBS-EOD had the same GBS strains. In 32 cases of GBS-EOD, the top three serotypes were III, Ia, and Ib, while the top three genotypes were ST17, ST23, and ST19. ST17 was the dominant genotype of serotype III, which was the most common prevalent in GBS-EOD [72.2% (13/18)], and ST23 was the dominant genotype of serotype Ia, the second most prevalent in GBS-EOD [87.5% (6/8)]. There were statistically significant differences in serotypes (*p* = 0.046) and genotypes (*p* = 0.000) distribution between the 266 pregnant women without GBS-EOD neonates and 32 cases of GBS-EOD.

**Conclusion:** This study revealed a statistically significant associations of GBS serotype Ia, and ST17 and ST23 between GBS colonization in women during late pregnancy and in neonatal GBS-EOD. The GBS ST23 of serotype Ia and ST17 of serotype III possessed a strong pathogenicity.

## Introduction

Group B *Streptococcus* (GBS) is one of the important gram-positive pathogens causing invasive perinatal neonatal infection (1). GBS can produce a number of important virulence factors, such as the capsular polysaccharide and a pore-forming toxin, β-hemolysin (2). Clinically, GBS is a conditional pathogen mainly colonized in the genitourinary system and the gastrointestinal tract of pregnant women, with an ~10–35% colonization rate (1). However, only 1–2% of these pregnancies result in neonatal invasive GBS infection, known as early-onset GBS disease (GBS-EOD), possibly through the placenta, amniotic fluid, or the birth canal (3). GBS-EOD includes severe pneumonia, sepsis, and meningitis (3). GBS is the leading cause of neonatal bacterial infection during gestation and after delivery, with significant mortality rates in premature infants (3). In China, several studies have reported 0.55–1.79 cases of GBS-EOD per 1,000 live births and severe GBS infection cases (4, 5). Vaginal GBS colonization in pregnant women is an important risk factor for neonatal GBS-EOD (6, 7). Thus, maternal GBS screening and subsequent intrapartum antibiotic prophylaxis (IAP) or even vaccination could effectively prevent neonatal GBS-EOD (3). However, since high-level coordinated health care management is not available in developing countries (8); thus, vaccination could be considered as a practical approach to prevent GBS-EOD (9) and is currently under development (10). Serotyping and genotyping of virulent GBS types could help clinicians identify high-risk pregnant women or infants for monitoring and treatment. A better understanding of virulent GBS prevalence could help identify GBS genotypes for developing specific vaccines.

To date, there are 10 known GBS serotypes (Ia, Ib, and II-IX) that are categorized based on capsular polysaccharide (CPS) composition (11). The known GBS gene sequence type (ST) contains more than 1,000 species, and two-thirds of the clinically detected GBS strains include ST-1, ST-17, ST-19, and ST-23 (12). However, previous studies have confirmed regional and ethnic variations in GBS infection and in serotype and genotype distribution in pregnant women (13). Different GBS serotypes and genotypes in pregnant women lead to differences in pathogenicity and hazards to neonatal GBS-EOD (14). Thus, in this study, we serotyped and genotyped GBS in samples from late pregnant women and neonatal GBS-EOD. Our findings provide a reference for future prevention and treatment of perinatal GBS infection in neonates. We also expect that our findings will add useful information for GBS vaccine development in China.

## Materials and Methods

### Study Populations

**Figure 1:**
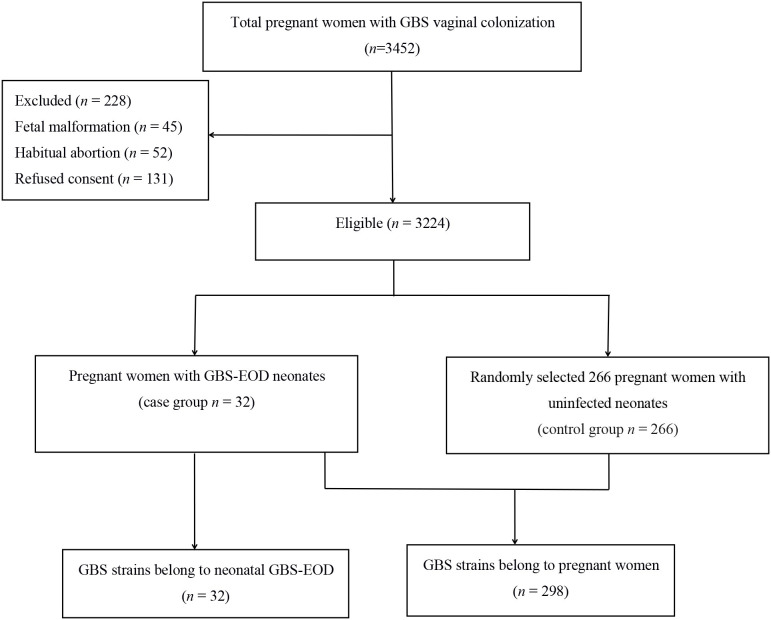
Flow chart of case and control enrollment and selection.

This study was approved by the Ethics Committee of the Women and Children's Hospital, Xiamen University (Xiamen, China). All participants or their guardians provided written informed consent before enrolling in this study. A total of 3,452 pregnant women with GBS colonization during late pregnancy (35–37 weeks gestation) were admitted to our hospital for labor between June 2016 and June 2018, while 228 cases were excluded according to our study criteria (Figure 1), resulting in 3,224 pregnant women enrolled into this study. The average age of 3,224 pregnant women was 28.6 ± 3.8 years old. Thirty-two neonates of their offsprings developed invasive GBS-EOD and hospitalized in the neonatology department of our hospital. The GBS-EOD diagnosis was determined according to the criteria of the Practical Neonatology (Fifth Edition) (15). These invasive GBS-EOD included GBS pneumonia, sepsis, and meningitis. GBS-EOD cases were excluded for: (1) positive GBS culture of the upper airway sputum or body surface secretion, but without any clinical manifestations; (2) neonates with congenital malformations; and (3) neonates with genetic metabolic diseases. These 32 GBS-EOD cases included 18 males and 14 females with an average age of 5 h (1.00, 14.25), average gestational age of 38.3 ± 3.3 weeks, average birth weight of 3,036.0 ± 772.6 g, and IAP usage rate of 3.1% (1/32) among mothers.

We then statistically calculated the sample size. GBS colonization rate of pregnant women in our hospital was 14.5% based on a previous study (6) and the allowable error δ was 4%. If α = 0.05, the required sample size could be 298 cases. Among the enrolled 3,224 pregnant women with GBS colonization during late pregnancy, 32 cases of colonization (0.99%) caused neonatal GBS-EOD (case group), while 266 GBS strains were randomly selected from pregnant women with uninfected neonates (control group). Thus, a total of 298 GBS strains from pregnant women and 32 strains from neonates with GBS-EOD were enrolled for serotyping and genotyping (Figure 1), indicating sufficient statistical power for this study.

### GBS Culture

Pregnant women at 35–37 weeks of gestation submitted samples for GBS bacterial culture collected by swabbing both the lower vagina and rectum (through the anal sphincter) into the plastic TranSwab device (Qi Xing Biotechnology Company, Suzhou, China). The swabs were then cultured in the plastic tube at 35°C in an incubator containing 5% CO_2_ for 24–48 h. The chromogenic agar in the tube undergoes color change in the presence of β-hemolytic GBS colonies. The positive tubes were then selected and cultured in a Colombian blood agar plate (Beiruite Biotechnology Co. Ltd., Zhengzhou, China) for further bacterial identification and determination of the GBS strains using the American PHOENIX100 bacterial identification system (Becton and Dickinson Company, Franklin Lake, NJ, USA). Tracheal secretion, blood samples, and the cerebrospinal fluid were also collected from neonates born to the enrolled pregnant women, who were subsequently admitted into the neonatal ward, and subjected to the GBS detection. All strains were stored in a −80°C freezer.

### GBS Serotyping

All 298 strains from mothers and 32 strains from neonates were confirmed as having GBS, and the isolates were serotyped using the latex agglutination kits for GBS Ia, Ib, and II–IX (Statens Serum Institut, Copenhagen, Denmark) according to the manufacturer's protocols. The isolate was defined as non-typeable (NT) when it could not be categorized into any serotype (16).

### Multi-Locus Sequence Typing (MLST) of GBS Strains

Chromosomal DNA was extracted from overnight cultures of the isolates at 35°C on 5% Müeller-Hinton agar using a DNA Mini Kit (Qiagen, Hilden, Germany) according to the manufacturer's instructions. The DNA samples were then subjected to PCR-amplification of seven housekeeping genes (*adhP, pheS, atr, glnA, sdhA, glcK*, and *tkt*) using oligonucleotide primers according to a previous study (16). The resulting PCR products were DNA-sequenced by Shenzhen Huada Gene Technology Co. Ltd. (China). The data were then submitted to the GBS MLST database (http://pubmlst.org/sagalactiae/info/primers.shtml) and the Chromas software and the MLST database (http://pubmlst.org/sagalactiae) were utilized to assign alleles at their seven loci, while each isolate was defined using the sequence type (ST) accordingly (16).

### Statistical Analysis

SPSS statistics for Windows, Version 25.0 (IBM Corp., Armonk, NY, USA) was used to statistically analyze our data. The qualitative variables were compared using the chi-square or Fisher's exact test, while the correlation analysis was presented by the coefficient of the contingency. Analysis of multiple sample rates was performed using the *post-hoc* test and the difference was judged according to the adjusted standardized residual. Statistical significance was determined by an absolute value of the normalized residual that was >2 or the difference between the observed frequency and the expected frequency value. A *p* < 0.05 was considered statistically significant.

## Results

### Distribution of GBS Strain Serotype and Genotype in 266 Pregnant Women

Among the 298 GBS isolates, we obtained 266 strains of GBS in the pregnant women without GBS-EOD neonates and 32 GBS strains with GBS-EOD neonates. We also found that these 32 mothers and their offsprings with GBS-EOD had the same GBS strains, including the same serotype and genotype. There were 8 serotypes among 266 GBS isolates. The most predominant serotype was III, accounting for 54.9% (146/266) of all isolates, followed by Ib [17.3% (46/266)], V [10.1% (27/266)], Ia [9.4% (25/266)], II [4.9% (13/266)], NT [1.0% (3/266)], VI [0.8% (2/266)], VIII [0.8% (2/266)], and IX [0.8% (2/266)].

There were a total of 42 STs identified among these 266 GBS isolates and ST19 was the most prevalent type [19.9% (53/266)], followed by ST862 [9.4% (25/266)], ST12 [7.9% (21/266)], ST17 [7.5% (20/266)], ST10 [7.1% (19/266)], ST23 [4.9% (13/266)], ST131 [4.1% (11/266)], and ST651 [3.8% (10/266)]. The remaining 34 genotypes were a single digit, and six strains were undetermined.

Furthermore, the main genotype of the serotype Ia was ST23 [28.0% (7/25)], while serotype Ib was dominated by ST12 [41.3% (19/46)] and ST10 [28.3% (13/46)], and serotype III was mainly comprised of ST19 [30.8% (45/146)] and ST17 [13.7% (20/146)]. We also observed unusual associations among these serotypes; serotype Ia with ST4 [16.0% (4/25)] and ST221 [16.0% (4/25)], serotype Ib with ST339 [6.5% (3/46)], and serotype III with ST862 [15.1% (22/146)] and ST862 [6.8% (10/146)]. However, the main serotype of ST17, ST19, ST131, ST651, and ST862 was serotype III [100.0% (20/20), 84.9% (45/53), 90.9% (10/11), 90.0% (9/10), and 88.0% (22/25), respectively], while ST10 and ST12 were associated with serotype Ib [73.7% (14/19) and 90.5% (19/21)], ST23 was associated with serotype Ia [53.8% (7/13)], and ST1 was associated with serotype V [62.5% (5/8)].

### Distribution of GBS Strain Serotype and Genotype in 32 Neonatal GBS-EOD

There were a total of five serotypes in the 32 cases of neonatal GBS-EOD. We identified 18 strains (56.3%) as serotype III, 8 strains (25.0%) as serotype Ia, 3 strains (9.4%) as serotype Ib, 2 strains (6.2%) as serotype II, and 1 strain (3.1%) as serotype V.

The DNA ST analysis revealed 10 STs among these 32 GBS isolates. ST17 was the most prevalent type [43.8% (14/32)], followed by ST23 [18.8% (6/32)], ST19 [12.5% (4/32)], and ST12 [6.3% (2/32)]. However, other genotypes, such as ST1, ST10, ST24, ST27, ST131, and ST651, were also identified, but there was only one isolate detected for each ST [all 3.1% (1/32)].

In these 32 cases of neonatal GBS-EOD, the top three serotypes were III, Ia, and Ib. The dominant and most prevalent genotype of serotype III was ST17 [72.2 % (13/18)]; the dominant genotype with the second most prevalent serotype Ia was ST23 [87.5% (6/8)]; and the dominant genotype with the third most prevalent serotype Ib was ST12 [66.7% (2/3)]. With regard to genotypes, the top three were ST17, ST23, and ST19 in these 32 cases of neonatal GBS-EOD. The dominant and most prevalent serotype of ST17 was III [92.9% (13/14)]; the second most prevalent serotype of ST23 was Ia, and the third more prevalent serotype of ST19 was III (Table 1).

**Table 1:** Genetic diversity of five GBS serotypes in 32 strains of neonatal GBS-EOD.

	***N***	**ST1**	**ST10**	**ST12**	**ST17**	**ST19**	**ST23**	**ST24**	**ST27**	**ST131**	**ST651**
Ia	8	1	0	0	0	0	6	0	0	0	1
Ib	3	0	0	2	0	0	0	0	0	1	0
II	2	0	1	0	1	0	0	0	0	0	0
III	18	0	0	0	13	4	0	0	1	0	0
V	1	0	0	0	0	0	0	1	0	0	0

### Association of Clinical Diagnosis and GBS Serotypes and Genotypes in 32 Neonatal GBS-EOD

There were only three types of clinical diagnoses in the 32 cases of neonatal GBS-EOD, including 13 cases of pneumonia (one death with serotype Ia and ST23), 14 cases of sepsis, and 5 cases of meningitis (one death with serotype III and ST17). These were the main clinical diagnoses rather than including only diagnosis based on clinical manifestations. Among these pneumonia, sepsis, and meningitis diagnoses, serotype III was the main GBS type, accounting for 46.2% (6/13), 50% (7/14), and 100% (5/5), respectively, although there was no statistically significant difference in distribution of the five GBS serotypes in these three diagnoses in GBS-EOD (*p* = 0.654; Table 2). Among these three diagnoses, ST17 was dominant, accounting for 5/13 cases (38.5%) in pneumonia, 5/14 cases (35.7%) in sepsis, and 4 /5 cases (80.0%) in meningitis. However, there was no statistically significant difference in distribution of the 10 GBS genotypes in the three diagnoses in GBS-EOD (*p* = 0.752; Table 3).

**Table 2:** Serotype distribution of 32 GBS-EOD cases with pneumonia, sepsis, and meningitis [*n* (%)].

**Disease**	***N***	**Ia**	**Ib**	**II**	**III**	**V**
Pneumonia	13	3 (23.1)	2 (15.4)	1 (7.7)	6 (46.1)	1 (7.7)
Sepsis	14	5 (35.7)	1 (7.1)	1 (7.1)	7 (50.0)	0 (0)
Meningitis	5	0 (0)	0 (0)	0 (0)	5 (100.0)	0 (0)
χ^2^-value	–					
*P-*value	0.654^*^					

* *Fisher's exact test*.

**Table 3:** Genotype distribution of 32 GBS-EOD cases with pneumonia, sepsis, and meningitis [*n* (%)].

**Disease**	***N***	**ST1**	**ST10**	**ST12**	**ST17**	**ST19**	**ST23**	**ST24**	**ST27**	**ST131**	**ST651**
Pneumonia	13	1 (7.7)	0 (0)	0 (0)	5 (38.4)	2 (15.4)	2 (15.4)	0 (0)	1 (7.7)	1 (7.7)	1 (7.7)
Sepsis	14	0 (0)	1 (7.1)	1 (7.1)	5 (35.7)	2 (14.4)	4 (28.6)	1 (7.1)	0 (0)	0 (0)	0 (0)
Meningitis	5	0 (0)	0 (0)	1 (20.0)	4 (80.0)	0 (0)	0 (0)	0 (0)	0 (0)	0 (0)	0 (0)
χ^2^-value	–										
*p-*value	0.752^*^										

* *Fisher's exact test*.

### Association of Serotype and Genotype Between Case and Control Groups

This cohort of patients and neonatal GBS-EOD was divided into case and control groups according to the groups of pregnant women with and without GBS-EOD neonates. We then compared GBS serotypes between these two groups (Table 4) and found that GBS serotypes V, VI, VIII, IX, and NT did not cause GBS-EOD (only one case in serotype V), and these were classified as the remaining serotypes. The data showed a statistically significant difference in the serotypes compared to the remaining serotypes (*p* = 0.046) between case and control groups with a column contact number of 0.183. A further analysis showed that the adjusted absolute value of standardized residuals of serotype Ia was the largest (>2) and the difference was statistically significant, whereas serotype III was only 0.1 and there was no statistically significant difference (Table 4). In terms of GBS genotype, there were 32 genotypes and undetermined genotypes that did not cause GBS-EOD; these were classified as the remaining genotypes. We found a statistically significant difference (*p* = 0.000) in disease-related genotypes compared to the remaining genotypes between case and control with a column contact number of 0.430 (Table 5). A further analysis showed that the adjusted standard residuals of ST17 and ST23 genotypes had the largest absolute values (6.1 and 3.0, respectively), indicating that they had a strong pathogenicity, whereas the remaining genotypes had a standardized residual value of −4.2, suggesting less pathogenicity (Table 5).

**Table 4:** Association of vertical transmission of GBS serotypes between case and control groups.

**Groups**	***N***	**Ia**	**Ib**	**II**	**III**	**The remaining serotypes**				
						**V**	**VI**	**VIII**	**IX**	**NT**
Case group	32	8 (2.7)^*^	3 (−1.1)	2 (0.3)	18 (0.1)			1 (−1.7)		
Control group	266	25 (−2.7)	46 (1.1)	13 (−0.3)	146 (−0.1)			36 (1.7)		
χ^2^-value					–					
*p-*value					0.046					

* *Case (standardized residuals after adjustment)*.

**Table 5:** Association of vertical transmission of GBS genotypes between case and control groups.

**Group**	***n***	**ST1**	**ST10**	**ST12**	**ST17**	**ST19**	**ST23**	**ST24**	**ST27**	**ST131**	**ST651**	**The remaining genotypes**
Case	32	1 (0.0)^*^	1 (−0.9)	2 (−0.3)	14 (6.1)	4 (−1.0)	6 (3.0)	1 (0.3)	1 (0.2)	1 (−0.3)	1 (−0.2)	0 (−4.2)
Control	266	8 (0.0)	19 (0.9)	21 (0.3)	20 (−6.1)	53 (1.0)	13 (−3.0)	6 (−0.3)	7 (−0.2)	11 (0.3)	10 (0.2)	98 (4.2)
χ^2^-value	51.34											
*p-*value	0.000											

* *Case (standardized residuals after adjustment)*.

## Discussion

In the current study, we found that serotype III was the most commonly distributed serotype in 266 GBS strains in pregnant women without GBS-EOD cases, accounting for 54.9%, followed by Ib, V, and Ia. A previous multicenter study in Shanghai showed that GBS serotypes III, V, and Ia were the most important serotypes in pregnant women with GBS colonization, accounting for 79.7% (17); thus, our current data (74.4%) are consistent with the previous data. A recent worldwide review of GBS isolates from pregnant women also showed that 85% of GBS serotypes were Ia, Ib, II, III, and V (18). These GBS serotypes accounted for 96% of GBS strains in the United States and 93% in Europe. In our current study, these five serotypes occurred in 96.6% of the total isolated GBS strains. Furthermore, we found that GBS serotype Ib was the second most prevalent serotype, slightly increased compared to that of Shanghai's study, but similar to that of the United States and European data (17, 18). Studies from other regions also indicated that serotype III was predominant in pregnant women (19, 20).

Our current study also assessed the genotype distributions of 266 GBS strains isolated from pregnant women and identified a total of 42 STs and 6 undetermined (UD) STs, with ST19 being the most prevalent (19.9%), followed by ST862, ST12, ST17, ST10, ST23, ST131, and ST651. Among these, ST1, ST17, ST19, and ST23 accounted for 35.3% of total GBS genotypes, which was inconsistent with that of a previous Canadian study reporting only 66.7% (12). The reason for this discrepancy may be due to the variance and dispersion of genotypes in different populations of studies. Furthermore, several previous Chinese epidemiological studies reported that GBS ST19 was the most prevalent genotype in GBS serotype III (72.7 and 75.0%) (21, 22). Our current data showed that the main genotypes were ST19 (45/146 cases, 30.8%) and ST17 (20/146 cases, 13.7%) in GBS serotype III, which was consistent with the previous studies (21, 22). Our data also demonstrated that GBS ST19, ST17, ST131, ST651, and ST862 were associated with serotype III, while ST23 was mainly serotype Ia. Serotype III was mainly associated with ST19 and ST17, and serotype Ia was mainly associated with ST23, consistent with the previous studies (23, 24), indicating that there are dominant genotypes and serotypes in pregnant women.

Interestingly, our current study also showed that serotypes III and Ia were the predominant serotypes in the 32 GBS strains in neonatal GBS-EOD, accounting for 81.3% of all serotypes. This indicates that these two serotypes may possess the strongest virulence for GBS-EOD. Indeed, a previous study revealed that GBS serotypes III and Ia were more virulent in causing GBS maternal colonization or infection and neonatal morbidity for 50% of the cases (23). However, we also found that pregnant women with GBS serotypes VI, VIII, IX, and NT did not induce GBS-EOD in neonates, while GBS serotype IV and VII did not occur in pregnant women, indicating these six GBS serotypes were harmless. Moreover, among these 32 cases of neonatal GBS-EOD, sepsis was the most common diagnosis, accounting for 14/32 (43.8%) cases, while pneumonia accounted for 13/32 (40.6%) cases and meningitis 5/32 (15.6%) cases, similar to a previous study (25). Further analysis showed that the main GBS serotype was III, regardless of GBS-induced pneumonia, sepsis, or meningitis, followed by serotype Ia. Serotype III accounted for 100% of early-onset meningitis of GBS. A previous study also showed that neonatal GBS meningitis was significantly associated with GBS serotype III (26). In addition, we found 10 STs in these 32 cases of GBS-EOD and ST17, ST23, and ST19 together accounted for 75.0% (24/32) of the total isolates, while ST17 associated with serotype III accounted for 80.0% (4/5) of the meningitis cases. These data agree with previous studies (23, 27) reporting that GBS serotype III in ST17 was more hypervirulent and led to higher meningitis cases. The serotypes of the death cases were Ia and III and the genotypes were ST17 and ST23 in GBS-EOD.

In addition, our current study revealed an association of GBS serotypes between pregnant women and neonatal GBS-EOD with an adjusted standardized residual of serotype Ia as 2.7 (>2) and serotype III as 0.1, while the vaginal colonization of serotype III was the most common (55.0%) and the vertical transmission rate was low, consistent with previous studies (28, 29). In contrast, the vaginal colonization of serotype Ia was low (11.1%), whereas the capability of vertical transmission was stronger, which was more likely to result in neonatal GBS-EOD. From this point of view, GBS serotype Ia virulence was stronger than that of serotype III. Moreover, there was a strong association of genotypes between pregnant women and neonatal GBS-EOD, especially the adjusted absolute residual values of ST17 and ST23 of 6.1 and 3.0, respectively. These two genotypes had the strongest capacity in vertical transmission and causing neonatal GBS-EOD. Further study will validate our current finding *in vitro*.

Our current study was retrospective and contained a limited number of study subjects. Specifically, we only included GBS-EOD cases and we had a limited study population from only one medical center and one regional hospital in Xiamen, China.

## Conclusions

In summary, our current data demonstrated that there is an association of GBS serotype Ia and genotypes ST17 and ST23 colonized in late pregnancy and GBS-EOD in neonates. The ST23 of serotype Ia and ST17 of serotype III possessed a strong hypervirulence to cause GBS-EOD. Further studies are needed to confirm the application of these GBS serotypes and genotypes for future treatment and development of vaccinations against GBS infection and colonization.

## Data Availability Statement

All datasets generated for this study are included in the article.

## Ethics Statement

This study was approved by the Ethics Committee of the Women and Children's Hospital, Xiamen University (Xiamen, China). All participants or their guardians provided written informed consent before enrolling in this study.

## Author Contributions

LX and CC conceptualized and designed the study, reviewed, and revised the manuscript. ZY searched the literature, performed the data analyses, and prepared the manuscript. WJ, ZX, and CL carried out experiments and analyzed experimental data. HM, MS, and LY collected data. All authors read and approved the final version of the manuscript.

## Conflict of Interest

The authors declare that the research was conducted in the absence of any commercial or financial relationships that could be construed as a potential conflict of interest.
